# Computational complexity as a potential limitation on brain–behaviour mapping

**DOI:** 10.1111/ejn.16636

**Published:** 2025-01-07

**Authors:** Ayberk Ozkirli, Michael H. Herzog, Maya A. Jastrzębowska

**Affiliations:** ^1^ Laboratory of Psychophysics Brain Mind Institute, École Polytechnique Fédérale de Lausanne (EPFL) Lausanne Switzerland; ^2^ Department of Education and Psychology Freie Universität Berlin Berlin Germany

**Keywords:** brain–behaviour mapping, cognitive ontology, computational complexity, localisationism, neural degeneracy

## Abstract

Within the reductionist framework, researchers in the special sciences formulate key terms and concepts and try to explain them with lower‐level science terms and concepts. For example, behavioural vision scientists describe contrast perception with a psychometric function, in which the perceived brightness increases logarithmically with the physical contrast of a light patch (the Weber‐Fechner law). Visual neuroscientists describe the output of neural circuits with neurometric functions. Intuitively, the key terms from two adjacent scientific domains should map onto each other; for instance, psychometric and neurometric functions may map onto each other. Identifying such mappings has been the very goal of neuroscience for nearly two centuries. Yet mapping behaviour to brain measures has turned out to be difficult. Here, we provide various arguments as to why the conspicuous lack of robust brain–behaviour mappings is rather a rule than an exception. First, we provide an overview of methodological and conceptual issues that may stand in the way of successful brain–behaviour mapping. Second, extending previous theoretical work (Herzog, Doerig and Sachse, 2023), we show that brain–behaviour mapping may be limited by complexity barriers. In this case, reduction may be impossible.

## Graphical abstract

Robust one‐to‐one mappings between the sensory stimulus and the behavioural level (top and bottom) do not imply that mappings can be found between the behavioural and the neural levels or the stimulus and neural levels, that is, within the inverted hourglass. For this reason, reduction from behaviour to brain processes to chemical and physical processes may be impossible.

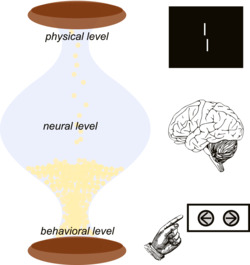

**Abbreviations**
2AFC: two‐alternative forced‐choice
ANN: artificial neural network
BOLD: blood‐oxygen‐level‐dependent
GM: grey matter
IT: inferotemporal cortex
PCA: principal component analysis
SVM: support vector machine
V1: primary visual cortex
V2: secondary visual cortex

## 1 HISTORICAL OVERVIEW OF BRAIN–BEHAVIOUR MAPPING

A fundamental goal of cognitive neuroscience is to understand the link between the brain on one side and perception and cognition, observed through behaviour, on the other side—what we refer to here as *brain–behaviour mapping*. The debate surrounding the way in which behaviour maps to the brain predates modern experimental methods. Nearly two centuries ago, Franz Joseph Gall put forward the view that the cortex is made up of ‘cerebral organs’, each corresponding to one of 27 distinct mental faculties (Gall, 1835; McCaffrey, 2023). Gall's organology was a strong form of localisationism, linking functions like ‘love of offspring’, ‘wit’ or ‘memory for locations’ with head bumps and other cranial features (Eling et al., 2017). Meanwhile, Marie Jean Pierre Flourens proposed the antithetical idea of equipotentialism, in which all areas of the brain would contribute to all different kinds of function (Flourens, 1842; McCaffrey, 2023).

**FIGURE 1:**
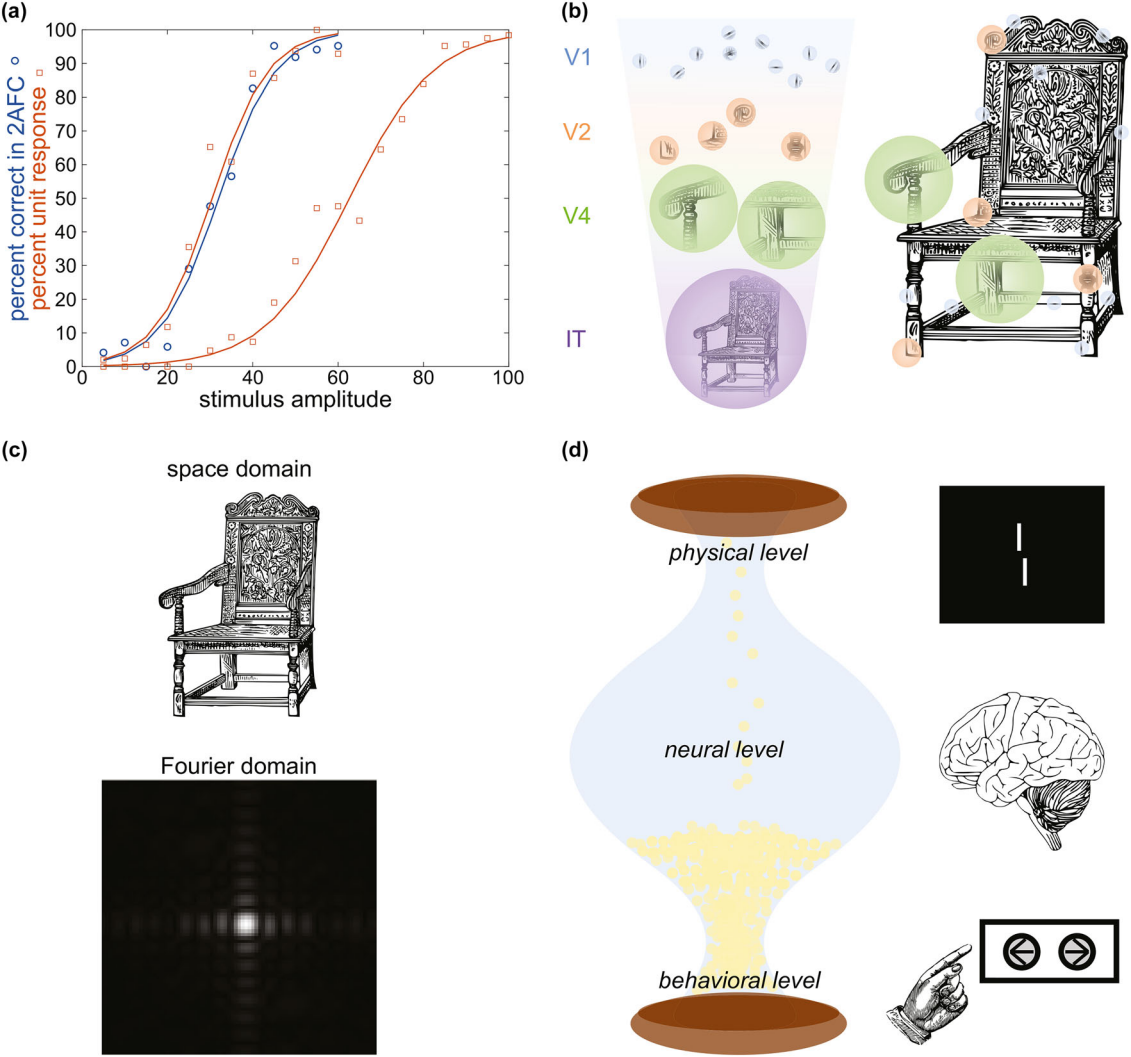
(a) A hypothetical example of a psychometric function and the corresponding neuronal response (neurometric) function. As stimulus amplitude increases (*x*‐axis), detection improves, as evidenced by the logistic growth of the percent correct in a two‐alternative forced‐choice (2AFC) task (*y*‐axis, blue circular data points). The probability of the neuron that is selective to the stimulus responding follows a very similar function, as seen in the logistic curve of the percent unit response (*y*‐axis, orange square data points). In another neuron that is less selective for the given stimulus, detection is much weaker, requiring a larger stimulus amplitude to saturate to the maximal response (100%). Inspired by Figure 6 in Parker and Newsome (1998). (b) A caricature of the subpart coding framework. Low‐level features of the chair (lines) are processed in primary visual cortex (V1), more complex features (angles) are processed in secondary visual cortex (V2), shapes are processed in visual area V4, and the entire chair object is represented in inferotemporal cortex (IT). (c) Fourier transforms of the chair in B ‘coded’ by spatial frequencies rather than localised spatial features. (d) Inverted hourglass architecture of brain–behaviour mapping: an illustration of the relationship between the low‐dimensional physical level (e.g. a Vernier stimulus with the bottom line offset to the left or right of the top line), the high‐dimensional neural level (over 140 million neurons in V1 alone), and the low dimensional behavioural level (e.g. a 2AFC task to determine the Vernier offset direction).

The nature of the empirical approaches used to study brain–behaviour mappings has arguably led to an entrenchment of localisationist rather than equipotentialist thinking. Early lesion deficit studies supported the view of a one‐to‐one mapping between brain and behaviour (Genon et al., 2018; Genon et al., 2022; Price & Friston, 2002), for example with the seminal discoveries of Broca's and Wernicke's language‐specialised regions (McCaffrey, 2023). Neurophysiology studies followed in this vein, with attempts to map psychometric functions to neurometric functions (Figure 1a). Landmark findings linking neuronal responses to specific perceptual properties like orientation (Hubel & Wiesel, 1962), sound frequency (Kiang, 1990) or motion detection (Dubner & Zeki, 1971; Hubel & Wiesel, 1962) added support to the notion of functional localisation even at the level of individual neurons.

The advent of modern noninvasive neuroimaging spurred neuroscientists to link localised brain measures—for example cortical thickness, surface area, grey matter volume and blood‐oxygen‐level‐dependent (BOLD) signal change—with experimentally derived behavioural measures (Genon et al., 2022; Kanai & Rees, 2011). For example, it was reported that political orientation correlates with grey matter volume, with greater liberalism associated with increased grey matter (GM) volume in the anterior cingulate cortex and greater conservativism coinciding with increased GM volume in the right amygdala (Kanai et al., 2011). However, a replication study of this and several other brain–behaviour mapping studies could not reproduce almost any of the findings, in fact finding evidence for the null hypothesis through confirmatory Bayesian hypothesis testing (Boekel et al., 2015). The failed replication initiated an extensive debate about replication issues (Boekel et al., 2016; Kanai, 2015; Muhlert & Ridgway, 2016). It became apparent that a concerningly large number of brain–behaviour mapping studies do not replicate (Button et al., 2013; Genon et al., 2022; Marek et al., 2022; Poldrack et al., 2017), calling localisationist approaches into question in general.

In this vein, recent years have seen a reevaluation of the theoretical framework underlying brain–behaviour mapping and the fundamental concept of functional localisation (Barack & Krakauer, 2021; McCaffrey, 2023; Mundale, 2002; Pessoa, 2022; Poeppel, 2012, 2017). While localisationist assumptions persist (often implicitly), there has been an explosion of tools that attribute the neural substrate of cognitive functions to widely distributed networks rather than spatially well‐separated nodes—for example, network neuroscience (Bassett & Sporns, 2017; Seguin et al., 2023), multivariate approaches such as representational similarity analysis (Kriegeskorte et al., 2008), and artificial neural networks (ANNs) as scientific models (Cichy & Kaiser, 2019; Doerig et al., 2023; Lindsay, 2021). Time will tell whether these new approaches will revolutionise our understanding of the way in which behaviour maps to the brain. Yet increasingly complex new techniques must go hand‐in‐hand with conceptual advancement to avoid the risk of scientists ‘reveal[ing] more and more about less and less’ (Buzsáki, 2020). In the following sections, we will examine possible theoretical explanations for the persisting lack of robust brain–behaviour mappings.

## 2 REASONS FOR FEW ROBUST BRAIN–BEHAVIOUR MAPPINGS

### 2.1 Cognitive and neural ontologies

Researchers seeking the neural correlates of cognitive functions rely on a certain categorical structure, which starts with abstract aspects of cognition (e.g. perception, attention, memory), subdivides them into finer constituent functions and defines the relations between them, resulting in a ‘parcellation of the mind’ or *cognitive ontology* (Poldrack, 2010; Price & Friston, 2005; Rust & LeDoux, 2023). For example, the classification of the Big Five personality traits was derived from large‐scale data collection from thousands of participants based on mathematical procedures such as principal component analysis (PCA). Hence, these traits are derived objectively. Still, the Big Five do not map robustly to brain structure (Avinun et al., 2020), at least according to prevailing neural ontologies that are based on measures such as cortical thickness, surface area, subcortical volume or white matter microstructure. Similarly, the integration of psychiatry and neuroscience has been notoriously difficult. In these cases, the difficulty in mapping may in part arise from the suboptimal way of categorising symptom combinations into clinical labels, which do not easily map onto brain features (Hyman, 2007). These failures may also arise due to the mismatch of abstraction levels between cognition and neuroscience—the so‐called ‘interface problem’ (Poeppel & Embick, 2005).

Another example comes from vision: Traditionally, visual illusions are categorised as illusions of size, contrast, orientation or texture. While such a categorisation makes sense according to the experimenter's high‐level understanding of visual properties, it has turned out that susceptibilities to different illusions within (and between) these categories do not correlate strongly with each other (Cretenoud et al., 2019; Grzeczkowski et al., 2017; Jastrzębowska et al., 2023), calling these classifications into question. It is, hence, not surprising to *not* find neural correlates for spatial illusions because there is nothing like spatial illusions in terms of a single process common to all spatial illusions in all humans. Importantly, for the sake of our discussion here, researchers have nevertheless tried to link subjective size perception to neural correlates and found a negative correlation between the surface area of primary visual cortex (V1) and size illusion magnitude in the Ebbinghaus, Ponzo and Delboeuf illusions (Moutsiana et al., 2016; Schwarzkopf et al., 2011; Schwarzkopf & Rees, 2013). However, these results could not be replicated (Jastrzębowska et al., 2023).

Up to now, we have considered one‐to‐one mappings between behaviour and the brain, as in the case of illusion magnitudes and V1 surface area. However, neuroscientists may want to map entire hierarchies onto each other. For example, according to the classic hierarchical view of the visual system, neurons in V1 code for basic components such as lines, intermediate areas represent more complex features, and higher areas like the inferotemporal cortex (IT) represent entire objects (Figure 1b). This framework assumes that the processing of the parts of an object is mirrored in the functional anatomy of the visual system. Metaphorically, the legs of a chair are processed in lower visual areas, which then project to higher visual areas, where the parts of the chair are combined, and the chair is processed in its entirety. We call this approach *subpart coding*. However, there is no reason to believe that coding can operate only in this way. Indeed, there are infinitely many other ways object recognition could work in the brain. For example, all processing may occur in Fourier space, from which the full object representations are decoded (Figure 1c). In this case, neural recordings from lower‐level areas would show no obvious correspondence to the subparts in the spatial domain as humans perceive them (the legs of the chair). In fact, the coding of neurons in ANNs used as vision models only partially reveals such subpart coding strategies, even though object recognition is as good as in humans (Lonnqvist et al., 2021; Xu & Vaziri‐Pashkam, 2021). Hence, the hierarchy of parts making up an object that makes sense in human terms may not necessarily be reflected by a corresponding hierarchy in the brain. Mathematically speaking, there are infinitely many orthonormal bases that can carry out the very same operations, but their respective representations are highly different.

In summary, the mapping between behavioural and cognitive constructs and neural processes depends heavily on how we define our cognitive and neural ontologies. So, what should we do? We could persist with the existing ones, hoping for sheer luck that the current ontologies work out or we might refine or reconsider our ontologies entirely to find new classifications.

### 2.2 Neural degeneracy

Degeneracy is ubiquitous in biology, which is even evident at the molecular level in the fact that distinct structures can produce the same output (many‐to‐one mapping). For instance, let us consider protein synthesis. The information found in an individual's DNA is first transcribed into RNA, where each triplet of nucleotides (a codon) codes for a particular amino acid. There are 64 possible codons (formed by combinations of four nucleotides A, U, C and G), while there are only 20 amino acids. This redundancy allows multiple codons to code for the same amino acid (i.e. codon degeneracy), which protects the system against mutations to a certain extent. For example, serine is not affected by mutations in the last nucleotide as it can be coded by four different RNA codons (UCU, UCC, UCA and UCG). Because proteins are made up of several amino acids, the same protein can be coded in multiple ways.

Increasing evidence for inter‐individual variability highlights the potential for degeneracy in neuroscience. In other words, humans vary substantially not only in their cognition, neural function and neuroanatomy but also in the mapping between these different levels, with distinct neuronal systems capable of carrying out the same functions (Edelman & Gally, 2001; Figdor, 2010; Price & Friston, 2002; Seghier & Price, 2018; Tononi et al., 1999). There are numerous well‐known examples of patients with significant portions of the brain missing (e.g., due to hydrocephalus) who functioned completely normally and only discovered their brain abnormalities incidentally.

It has been suggested that there are two types of neural degeneracy: within and between subjects (Noppeney et al., 2004). In the first case, a particular behaviour can be realised by different neural mechanisms within the same individual. In the second case, different individuals employ unique strategies to perform the same task, contributing to degeneracy at the population level. For instance, studies on working memory have shown that different brain regions are involved in the performance of the same task when individuals employ different task strategies (Pearson & Keogh, 2019; Sanfratello et al., 2014). Another study reported that, even within the same individual, there could be substantial variation in task strategy across different tasks measuring the very same thing (working memory, see (Morrison et al., 2016)).

These neurobiological considerations echo the multiple realisation argument (Block & Fodor, 1972; Figdor, 2010), which suggests that the same function can be mediated by different mechanisms across species. An everyday analogy would be the capacity of various operating systems (e.g. Windows, MacOS and Linux) to execute the same program (e.g. Microsoft Word). Applying this analogy to our point about inter‐individual degeneracy, one might say that each individual organism has its own unique operating system, which it can nevertheless use to carry out the same tasks.

## 3 REDUCTIONISM AND COMPLEXITY

As we have seen above, brain–behaviour mapping has not been very successful and we have given some examples as to why this may be the case, that is, localisationist assumptions, insufficient data or research methodology, improper cognitive ontologies or neural degeneracy. Yet another possibility is that reduction is not always possible due to what we call ‘complexity barriers’.

In philosophical terms, it seems that most neuroscientists subscribe (implicitly) to epistemological reductionism, that is, the view that we can reduce any perceptual phenomenon to neural mechanisms, which in turn can be explained by molecular processes, and so on, until we come up with an explanation in terms of particle physics. This is the position of physicalism, which states that everything is either physical or metaphysically connected to the physical (Stoljar, 2023). Expressed in terms of the standard model of particle physics, all matter is composed of fundamental particles—fermions and bosons, which mediate three of the four fundamental forces (the strong and weak nuclear forces, electromagnetism; additionally, there is gravity). In these terms, a human is not much different from a pineapple: both are composites of fermions. In addition, all biological processes and laws are nothing else than physical laws. For this reason, we should, at least in principle, be able to reduce cognitive processes to neurophysiological processes and in turn to basic physical processes. As mentioned earlier, this is exactly what neuroscientists try to do as laid out above. Following this idea, the job of neuroscience will be done when all perceptual and cognitive processes are explained in neural terms. However, even when one subscribes to physicalism, the existence of such reductive links does not imply that we can find them (Herzog et al., 2023).

Here is a real‐world example for what a complexity barrier may look like: Huntington's disease is a fatal neurodegenerative disorder. On the clinical level, there are clear‐cut symptoms, such as uncontrolled movements. On the genetic level, Huntington's disease is characterised by abnormally long repeats of three base pairs (CAG) on chromosome 4 (Walker, 2007). Clinical symptom severity and mortality are highly correlated with the number of CAG repeats, which vary strongly across the patient population. The more repeats there are, the earlier patients die. Hence, there are law‐like links between the genetic level and the clinical level. However, at the causally relevant neurobiological level in the cerebral cortex or striatum, one cannot reliably predict mortality or diagnose the disease. Until now, no meaningful correlations have been found. We only know that the causes must be somewhere in these two neural structures. Hence, reduction from clinical assessment to the genetic level via neuroscience is (at least currently) impossible because the number of neurons in the cerebral cortex and striatum makes up a complexity barrier. Whether or not this is an impenetrable barrier is an empirical question, and it may turn out that researchers are able to explain how Huntington's disease affects these regions in the future.

It can be shown mathematically that complexity barriers exist in principle, as long as the P ≠ NP assumption is true, meaning that while a solution to a problem is verifiable in *polynomial time*, it can only be found in *nondeterministic polynomial time*, making the problem *intractable*—that is, computationally infeasible to solve within a reasonable time as the problem size grows. The argument is as follows. In the example of Huntington's disease, we can see the causal pathway from the genetic level to clinical symptoms as a function, which is a concatenation of two functions, one from the genes to the brain and the second from the brain to motor behaviour. To learn the function, it seems that we just need to record sufficiently many samples from the brain. However, mathematical learning theory shows that no matter how many samples one observes, it is impossible to learn the function if the function is sufficiently complex. Importantly, this is only true for certain functions. For example, linear functions *f*(*x*) = *y* = *ax* + *b* can be determined by observing only two input–output pairs. For example, the input–output pairs (0, 1) and (3, 2) fully determine *f*, leading to *f*(*x*) = 1/3*x* + 1. However, for complex functions, such as the Boolean functions, one may observe as many pairs (*x*, *y*) as one wants and never learn the function, not even approximately (Herzog et al., 2023; Kearns & Valiant, 1994).

In fact, such complexity barriers are exploited for safe banking and for internet coding zillions of times every day. For example, when you send a message to a friend, the plaintext message is first encrypted on your side with a public key that is available to everyone. When the encrypted message (the ciphertext) is sent via the Internet, the only way to decrypt it is by using your friend's private key. Thus, only your friend can read the original message. All is accessible, except for the private key. If the same message is sent again, the coded message may look very different due to the randomness in the encrypting algorithm. Due to sufficient complexity, finding the original message is intractable unless one has the private key.

To better understand complexity and why it may create complexity barriers, let us consider a hypothetical example (Herzog et al., 2023). An animal always lifts its right limb when a red patch is presented and its left limb when a green patch is presented. Researchers take measurements from the animal's brain, which contains 60 binary neurons. Hence, each recording gives a vector with 60 entries of either 1 or 0 (the given neuron is either active or not). As in the cryptography example, at each presentation of the colour patch, the researchers see a new vector of neural responses (due to neuronal fluctuations). Researchers carry out as many experiments as they want, knowing which patch was presented, which vector of neural responses occurred, and which limb movement was carried out, the latter being fully determined by the colour of the patch. If coding is combinatorial, researchers will never find any pattern or rule in the large number of neural activation vectors. They will therefore be unable to characterise the link between the patch colour and vectors, on the one hand, or the vectors and the limb movement, on the other. Reduction is infeasible. The reason is that the state space of the neurons is 2^60^, which is a number larger than the number of seconds in the universe, counted from the time of the Big Bang. The key point is that it is possible to hide information in large state spaces. Even though the information is deterministic, it cannot be found because it is hidden in complexity. This is the essence of cryptography. For reference, the nervous system of the worm *Caenorhabditis elegans* has 302 neurons.

In the example above, it is crucial that the coding of neural activations is fully combinatorial, meaning that no subset of neurons (<60) would allow for decoding. For example, if the first entry of the vector were 1 for the red and 0 for the green patch consistently, decoding would be easy because entries 2 through 60 would be of no interest. One would only need to check the first neuron without having to search through all 2^60^ states.

## 4 DISCUSSION

Each scientific discipline comes with its own set of entities that shape its structure. For example, in personality research, the Big Five might serve as basic entities. In neuroscience, the neuron is the basic unit making up neural circuits, brain areas and networks. In vision research, understanding object recognition is potentially the ultimate goal and, hence, objects (lines, pineapples, faces, etc.) are the basic entities. Intuitively, researchers often assume that there must be a direct link between the entities of the various disciplines, such as between an object in the visual field and a neuronal circuit coding for it. Often more is expected, such that parts of an object map on lower‐level neurons, which project to higher level neurons coding for the entire object, that is, subpart coding. Hence, not only entities map but also entire hierarchies can be mapped. While this idea is intuitive, its current success is limited, with few robust brain–behaviour mappings identified. One possible reason for this limited success is that we need to change our ontologies to improve these mappings, either the cognitive or neurobiological ones, such as specific neural circuits or temporal firing patterns. Consider a successful example from classic biology: Whales and dolphins were once classified as fish because they live in the water like fish. However, this ontology did not align well with the tree of life, which in modern times corresponds to genetic proximity. Biologists replaced an ontology based on phenomenology with an ontology based on descendance. The whale is now a mammal—a classification that is not given by nature but by human definition and which may well change in the future.

Similarly, cognitive neuroscience may need to rearrange its entities or introduce new ones. In this debate, Buzsáki (2020) argues that cognitive neuroscience should develop its own terminology based on neural mechanisms, rather than relying predominantly on terms inherited from folk psychology (Buzsáki, 2020). Buzsáki advocates for an ‘inside‐out’ brain‐first approach, which entails using definable brain mechanisms as a starting point to define objective behaviours. However, Poeppel and Adolfi (2020) advocate a bidirectional approach, in which both brain‐first and behaviour‐first approaches mutually inform and constrain each other (Poeppel & Adolfi, 2020). Such an approach may eventually converge to the correct level of abstraction in both fields, making it more probable to find robust links between brain and behaviour.

Even if the cognitive and neural ontologies were defined in a way that could theoretically lead to robust brain–behaviour mappings, reduction is fundamentally challenged by neural degeneracy. Reduction aims for a one‐to‐one mapping between brain and behaviour, yet neural degeneracy illustrates the possibility of many‐to‐one mappings, both within and between subjects. In the brain of the same individual, distinct neuronal systems can fulfil an identical task. Similarly, the same function may be achieved in different ways in different individuals. While neural degeneracy provides evolutionary adaptability through flexibility and resilience against injury or variability, it complicates any straightforward mapping between brain and behaviour as one behaviour is not tied to a single, isolated neural pathway.

Assuming that there is no neural degeneracy and that ontologies are correctly defined, it may seem that brain–behaviour mapping can be easily achieved. However, as we have shown, there may be no simple links between scientific fields when complexity barriers exist. Such barriers exist in real life, such as in safe banking, where barriers are put in place to prevent unauthorised access. Similarly, in neuroscience, complexity barriers may be inherent to the inverted hourglass architecture of brain–behaviour mappings (Figure 1d), characterised by a low‐dimensional first level (the physical level), a high‐dimensional second level (the brain) and a low‐dimensional third level (behaviour).

Consider the example of an object categorisation task. Light is transformed into neural signals in the human retina, which has about 126 million photoreceptor cells (Molday & Moritz, 2015). The information is then projected to the visual cortex, which has approximately 5 billion neurons (Wandell et al., 2009). The output of one retinal photoreceptor is therefore analyzed by about 40 neurons in the visual cortex. The visual information is transformed into a decision‐relevant representation, which is then output as a behaviour in the object categorisation task. Hence, we have an inverted hourglass architecture with initial low‐dimensional input, expanded processing in the visual cortex, and then condensed outputs that manifest as behaviour (e.g. object categorisation). This architecture can hide the information about links between the input and output levels in complexity. For example, the distributed response patterns in early and intermediate visual cortex (V1 to human V4) do not differentiate between the exemplars of different categories, while later regions in ventral temporal cortex (like the fusiform face area or the parahippocampal place area) do (Grill‐Spector & Weiner, 2014). This is analogous to the cryptography example: The sensory input is the plaintext message, the neural signals in the visual cortex correspond to the ciphertext, and the observed behaviour is the decrypted output. However, researchers cannot decode the category from intermediate visual processing stages as accurately as they can from semantic processing levels (Grill‐Spector & Weiner, 2014)—at least not at the moment. The category information is hidden in the complexity, yet the perceptual ‘ciphertext’ being transmitted through the neural signals is decrypted using the brain's ‘private key’, making category information accessible only from higher level stages of visual processing, where dimensionality decreases.

Complexity barriers also exist in ANNs, where the architecture and activity of neurons are fully known at each moment in time. Intuitively, this might suggest that understanding how the network functions should be straightforward. Now, imagine a researcher has trained an ANN to match human performance in a visual task and is attempting to reverse‐engineer the neural network to gain insights for vision science. For instance, the link between an input and its output is carried out by a subset of neurons in the network. Given the full observability and perturbability of the neural network, one might assume that the researcher could easily identify the smallest circuit needed to carry out the visual task (minimum sufficient circuit). However, Adolfi and colleagues have shown mathematically that this intuition is flawed because the system is so complex that finding the minimum sufficient circuit in the network is intractable (Adolfi et al., 2024). See Adolfi (2024) for a detailed discussion of complexity analytic approaches to cognitive science.

These findings highlight the fact that the search for reductive links from cognitive processes to neurobiological mechanisms may face complexity barriers due to the enormous computational time required. Given that the most advanced state‐of‐the‐art ANNs (e.g. Vision Transformer model with 22 billion parameters; Dehghani et al., 2023) are still less complex than the human brain (trillions of synaptic connections or ‘parameters’), it stands to reason that such an endeavour in the human brain is also intractable. Indeed, in line with our hypothetical animal example above, Ramaswamy (2019) has shown that the number of experiments required to establish a mechanistic link between neural activity and behaviour scales exponentially with the complexity of the circuit being studied, making it practically infeasible to find the link even for systems of modest size (Ramaswamy, 2019).

Here, we have argued that complexity barriers likely exist in systems with an inverted hourglass architecture (Figure 1d). Notably, there may be evolutionary reasons for such barriers to exist. The complexity observed in living organisms may have evolved to ‘hide’ the organism's crucial functioning from external exploitation, such as parasitic invasion or predation (Krakauer, 2017). For example, the parasite *Toxoplasma gondii* infects rodents (mice and rats). Infected mice exhibit reduced fear responses to cat odours, thus turning them into easy targets and allowing the parasite to enter its definitive host, the cat. While the parasite can hijack specific neural circuits to alter the rodent's behaviour, evolving complexity barriers could hinder the identification of these circuits and make it harder for the parasite to penetrate the relevant neural circuitry. Neural complexity can thus be understood metaphorically as an evolutionary cryptosystem, in which complex interactions and redundancies obscure direct mappings from physical stimulus, through neural activity, to behavioural outcomes.

Besides the evolutionary benefits, an inverted hourglass architecture may be advantageous from the perspective of neural coding and computational efficiency. Vision, for instance, is a complex pattern recognition problem, linking photoreceptor activations to object representations under varying conditions like changes in lighting, orientation and occlusion. Usually, such complex problems cannot be solved with simple linear approaches. Machine learning tools, such as support vector machines (SVMs), solve such problems by transforming the input space—analogous to ‘retinal’ input—into a higher dimensional space where the patterns can be linearly separated. This dimensionality expansion allows for the efficient classification of complex patterns that are not linearly separable in the original space. Similarly, the brain may utilise such dimensionality expansions to facilitate the separation and recognition of complex input patterns. For instance, by first expanding the dimensionality of neural representations during the encoding phase in the visual cortex, the brain can disentangle overlapping inputs, making their classification easier.

Complexity barriers may be impenetrable in brain–behaviour mapping, but it is not the end of neuroscience. As in the example of Huntington's disease, we can bypass these barriers in the brain by directly linking different levels, such as genetics and clinical diagnoses. In fact, most of neuroscience operates in this way, bypassing intermediate stages of processing for convenience. For example, vision research questions are studied in the brain regions of interest (e.g. V1) without consideration for the full causal pathway from the retina to this region. In short, we bypass complexity barriers in neuroscience research all the time—not just by directly linking the lower level (physical) with higher one (behaviour) but also by synthesising complex phenomena into ontologies and making simplifying assumptions about neural degeneracy. It is like in the drunkard's search: We search where the light is, not necessarily where the truth lies.

Future meta‐research may be able to determine whether a research question is too hard to answer—at least with currently available tools. We will know that reduction is possible only when we eventually achieve it. Conversely, we will never know that reduction is impossible. The considerations outlined above do not mean that brain–behaviour mapping is impossible, but it seems there is not necessarily a one‐size‐fits‐all procedure to derive explanations. Therefore, we may be better off embracing epistemic pluralism (Devezer et al., 2019; Krakauer et al., 2017; Rich et al., 2021).

## AUTHOR CONTRIBUTIONS

**Ayberk Ozkirli:** Conceptualization; writing — original draft. **Michael H. Herzog:** Conceptualization; writing — original draft. **Maya A. Jastrzębowska:** Conceptualization; visualization; writing — original draft.

## CONFLICT OF INTEREST STATEMENT

The authors declare no conflicts of interest.

### PEER REVIEW

The peer review history for this article is available at https://www.webofscience.com/api/gateway/wos/peer-review/10.1111/ejn.16636.

## Data Availability

This is a theoretical article so there is no relevant data.
